# Preparation and Characterization of Superabsorbent Polymers Based on Starch Aldehydes and Carboxymethyl Cellulose

**DOI:** 10.3390/polym10060605

**Published:** 2018-06-02

**Authors:** Jungmin Lee, Soohee Park, Hyun-gyoo Roh, Seungtaek Oh, Sunghoon Kim, Myounguk Kim, Donghyun Kim, Jongshin Park

**Affiliations:** 1 Department of Biosystems and Biomaterials Science & Engineering, Seoul National University, 1 Gwanak-ro, Gwanak-gu, Seoul 08826, Korea; archon04@snu.ac.kr (J.L.); ssangno@snu.ac.kr (H.-g.R.); bv206driver@snu.ac.kr (S.K.); 2 LOTTE Fine Chemical Co., Ltd., 19, Yeocheon-ro 217 beon-gil, Nam-gu, Ulsan 44714, Korea; shpark007@lottechem.com; 3 HYOSUNG CORPORATION, R&D Business Labs, 74, Simin-daero, Dongan-gu, Anyang-si, Gyeonggi-do 14080, Korea; ost@hyosung.com; 4 Korea Institute of Ceramic Engineering and Technology, Ceramic Fiber & Composite Center, Jinju 52851, Korea; myounguk@kicet.re.kr; 5 Korea Institute of Industrial Technology, Research Institute of Industrial Technology Convergence Human and Culture Convergence R&D Group, 143, Hanggaulro, Sangnok-gu, Ansan-si, Gyeonggi-do 15588, Korea; dhkim@kitech.re.kr; 6 Research Institute for Agriculture and Life Sciences, 1 Gwanak-ro, Gwanak-gu, Seoul 08826, Korea

**Keywords:** superabsorbent polymers, polysaccharides, carboxymethyl cellulose, starch aldehydes, citric acid

## Abstract

Superabsorbent polymers (SAPs) are crosslinked hydrophilic polymers that are capable of absorbing large amounts of water. Commercial SAPs are mostly produced with acrylic acid that cannot be easily biodegraded. Therefore, in this study, polysaccharide-based SAPs using carboxymethyl cellulose as a major component were prepared. Starch aldehydes and citric acid were selected due to their environment-friendly, non-toxic, and biodegradable properties compared to conventional crosslinking agents. Starch aldehydes were prepared by periodate oxidation, which forms aldehyde groups by taking the places of C–OH groups at C-2 and C-3. Furthermore, starch aldehydes were analyzed through the change in FT-IR spectra, the aldehyde quantitation, and the morphology in FE-SEM images. In the crosslinking of polysaccharide-based SAPs, the acetal bridges from starch aldehydes led to a large amount of water entering the network structure of the SAPs. However, the ester bridges from citric acid interfered with the water penetration. In addition, the swelling behavior of the SAPs was analyzed by the Fickian diffusion model and the Schott’s pseudo second order kinetics model. The relationship between swelling behavior and morphology of the SAPs was analyzed by FE-SEM images. In conclusion, polysaccharide-based SAPs were well prepared and the highest equilibrium swelling ratio was 87.0 g/g.

## 1. Introduction

Superabsorbent polymers (SAPs) are crosslinked hydrophilic polymers that are capable of absorbing large amounts of water [1]. It has been reported that the presence of hydrophilic groups, the flexibility of high polymer chain, and the availability of large free volumes among polymeric chains enhance the swelling capacity of SAPs [2]. Because of their excellent characteristics, SAPs are widely used in many products like disposable diapers, feminine napkins, agricultural and horticultural soil, gel actuators, water-blocking tapes, medicine for drug-delivery systems, and absorbent pads [3]. Commercially available SAPs are mostly produced with acrylic acid as a major component that cannot be easily biodegraded [4]. Therefore, bio-based SAPs utilized as a substitute for conventional synthetic polymers have been actively studied in recent years [5].

Polysaccharides are major sources of bio-based SAPs. There have been many studies using starch [4], carboxymethyl cellulose (CMC) [6], alginate [7], chitosan [8], and carrageenan [9]. Among these polysaccharides, starch is the most widely used material in preparing SAPs. Starch is a renewable and degradable polymer-obtained from various botanical sources [10]-composed of amylose, a linear polymer of α-(1-4) glucose units, and amylopectin, a linear polymer of α-(1-4) glucose units with periodic branches of α-(1-6) linkages [11]. Consequently, starch and its derivatives have been utilized in many industrial applications such as food, medicine, and cosmetic fields [12].

In SAP preparation, a chemical modification of starch to improve performance has been studied. Although there are many kinds of chemical modification, the most typical method is free radical graft copolymerization of vinyl monomers onto starch [13,14,15,16,17,18,19,20,21]. Except in a few cases [13], vinyl monomers used for copolymerization with starch are acrylic acid or acrylamide. Most grafting methods are solution processing in which water, starch, acrylic acid, initiator, and crosslinking agent are mixed and reacted while there are some cases of melt processing [14]. Other materials are added to improve the performance of the starch/acrylic acid copolymer. For example, small amounts of itaconic acid improve water absorption [17] while alginate increases pH sensitivity of the SAPs [18]. In addition, there have been studies conducted on preparing starch-based composite SAPs. To improve water absorption by improving the surface morphology of SAPs, zeolite and cellulose nanowhiskers are added [19,21].

Most starch/acrylic acid copolymers are advantageous in water absorption. However, as mentioned earlier, acrylic acid has poor biodegradability, which leads to environmental problems with their disposal [17,20]. From the cases where starch is degraded by α-amylase enzyme in starch/acrylamide SAPs [4,17], a certain disintegration of disposable SAPs by landfill can be expected [22], but complete biodegradation is not satisfactory. Therefore, in this study, polysaccharide-based SAPs using starch and CMC as main materials were prepared without acrylic acid. CMC is a major commercial derivative of cellulose and anionic water-soluble natural polymers, which are widely used in detergents and oil exploration as well as food, paper, and textile industries due to its viscosity-increasing properties [6,23]. Polar carboxyl groups render the cellulose chemically reactive and strongly hydrophilic, so the applications of CMC in SAP fields are very useful [24].

The addition of a crosslinking agent is also very important for SAP preparation. In this experiment, starch aldehydes and citric acid were selected as crosslinking agents. Starch aldehydes, along with their own absorptivity, were used for effective chemical bonding with the hydroxyl groups of CMC. In previous studies, based on the high functionality and reactivity of aldehyde groups, there have been attempts to use environment-friendly and non-toxic dialdehyde carbohydrates as crosslinking agents to replace glutaraldehyde. The linkages between aldehyde and hydroxyl groups are used to prepare films [25,26] while the linkages between aldehyde and amine groups are used to prepare films or gels and fix biological tissues [27,28,29]. Citric acid, which is environment-friendly, non-toxic, and biodegradable, is used as an excellent crosslinking agent in the food and drug industry [30,31]. Crosslinking goes through a mechanism where two carboxylic acid groups on citric acid form an anhydride through the loss of a water molecule. Then, this anhydride reacts with a hydroxyl group on the polysaccharide to form an ester bridge [32,33].

The experimental SAP preparation procedure consisting of starch aldehydes, CMC, and citric acid first involves modifying corn and potato starch to starch aldehydes by oxidation. In particular, the oxidation by sodium periodate changes the C-2 and C-3 hydroxyl groups to aldehyde groups [28,29] where the concentration of sodium periodate was varied in the oxidation process. The degree of substitution (DS) of starch aldehydes was confirmed by qualitative and quantitative analysis. Furthermore, the effects of starch aldehydes on preparation and water absorption behavior of the SAPs were analyzed. The water absorption performance of polysaccharide-based SAPs was also confirmed when citric acid was introduced.

## 2. Materials and Methods 

### 2.1. Materials

Corn and potato starch as well as sodium periodate were purchased from Samchun Pure Chemical, Co., Ltd., (Pyeongtaek, Korea). CMC (M_w_ = 700,000) was purchased from Acros Organics, (Belgium, WI, USA). Methanol used for washing as well as hydroxylamine hydrochloride, methyl orange, and sodium hydroxide used for DS calculation were purchased from Samchun Pure Chemical, Co., Ltd.(Pyeongtaek, Korea) Citric acid was purchased from Showa Chemical Industry Co., Ltd. (Tokyo, Japan) while sulfuric acid was used as the catalyst and purchased from Junsei Chemical Co., Ltd., Tokyo, Japan.

### 2.2. Preparation of Starch Aldehydes

A definite amount of sodium periodate was dissolved completely in 500 mL of distilled water at room temperature before 24.3 g of corn or potato starch were added. This mixture was charged into a three-necked flask fitted with an impeller. The vessel was heated to 40 °C under continuous stirring. After 16 h, a white slurry formed. Finally, this slurry was washed with distilled water and methanol before being dried for 24 h at 60 °C. The sample code and the detailed ingredients are shown in Table 1.

### 2.3. Preparation of Polysaccharide-Based SAPs

A total of 1 g of starch aldehydes (or native starch) and 1 g of CMC were added in 100 mL distilled water. The temperature of the flask was maintained at 100 °C for 1 h. After complete dissolution of the polysaccharides, 0.05 mL of sulfuric acid were added to the mixture and stirred for 5 min. At this time, citric acid was added to 10% or 20% molar ratio of anhydroglucose units in some samples. The obtained suspension was poured into a mold and dried in an air oven at 55 °C for 24 h. Finally, the film product was peeled from a mold and pulverized to prepare the powder. The sample code and the detailed ingredients are shown in Table 2.

### 2.4. FT-IR Spectroscopy

A FT-IR spectrometer (Nicolet iS5, Thermo Scientific, Waltham, MA, USA) was used to confirm the chemical structure of all samples. Before analysis, the samples were dried in an auto-desiccator (SANPLY DRY KEEPER, SANPLATEC Corp., Osaka, Japan) for 24 h. The samples were analyzed at a 4 cm^−1^ resolution with 32 scans between 650 and 4000 cm^−1^.

### 2.5. FE-SEM Analysis

A FE-SEM (AURIGA, Carl Zeiss, Oberkochen, Germany) was used to observe the morphology of all samples. Before analysis, the samples were dried in an auto-desiccator for 24 h. The specimens were coated with platinum and observed at 1000× magnification.

### 2.6. Quantitative Analysis of Starch Aldehydes

The aldehyde content of modified starch was determined according to a method described in literature [34]. First, 17.5 g of hydroxylamine hydrochloride was dissolved in 150 mL of distilled water before 6 mL of 0.05% methyl orange solution was added. This solution was diluted with distilled water up to 1 L volume and the pH was adjusted to 4.0. Then, 0.1 g of starch aldehydes was dissolved in 25 mL of hydroxylamine hydrochloride-methyl orange solution. The mixture was allowed to stand for 2 h and titrated with sodium hydroxide solution until the red-to-yellow end point was achieved.

### 2.7. Swelling Ratio Measurement

The tea bag method was used to measure the swelling ratio of the SAPs. A tea bag containing dried SAPs was immersed entirely in distilled water to reach equilibrium at room temperature. The swelling ratio was calculated by the following Equation (1):

Swelling ratio = (*W_STSAP_* − *W_ST_* − *W_ISAP_*)/*W_ISAP_* (1)

where *W_STSAP_* is the weight of the swollen tea bag containing swollen SAPs, *W_ST_* is the weight of the swollen tea bag only, and *W_ISAP_* is the initial weight of dried SAPs.

## 3. Results and Discussion

### 3.1. Oxidation of Starch and Preparation of Starch Aldehydes

**Figure 1:**
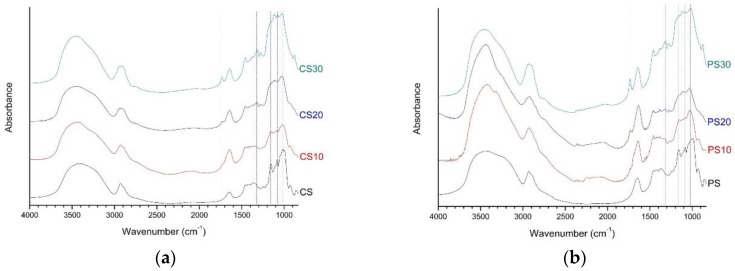
The FT-IR spectra of native starch and starch aldehydes: (**a**) corn starch series; (**b**) potato starch series.

Figure 1 showed the FT-IR spectra of native starch and starch aldehydes. In the spectra of native starch, strong and broad peaks were detected at 3600–3200 cm^−1^, which are due to hydrogen bonded hydroxyl groups [35]. In the detailed region of 1200–900 cm^−1^, there were several discernible peaks at 1156, 1081, 1018, and 929 cm^−1^, which are attributed to C–O bond stretching. The peaks at 1081 and 1018 cm^−1^ were the characteristics of C–O stretching in anhydroglucose ring [36]. The peak at 1645 cm^−1^ showed a bend of tightly absorbed water present in the starch. The bands at 2930 and 2890 cm^−1^ were the characteristics of C–H stretching [36,37].

A remarkable change in FT-IR spectra of starch aldehydes was the peak at 1732 cm^−1^, which is the most characteristic peak of C=O vibrations in aldehyde groups [29,38]. By increasing the sodium periodate content, the characteristic peak for C=O groups at 1315 cm^−1^ was increased, but the peaks at 1156 and 1081 cm^−1^ were weakened. In other words, three C–O bond peaks at 1156, 1081, and 1018 cm^−1^, which are the fingerprint regions of starch, gradually transferred into a broad band. It may be due to the fact that periodate oxidation mainly breaks the C–2 and C–3 bond of anhydroglucose units, followed by the formation of the aldehyde groups and the replacement of the C–OH groups at C–2 and C–3 [39,40]. In addition, the native starch peak at 929 cm^−1^ was weakened, but the new peak of starch aldehydes at 875 cm^−1^ was assigned to form hemiacetal bonds between aldehyde groups and neighbor hydroxyl groups [41].

### 3.2. DS Calculatoin of Starch Aldehydes

In the DS calculation by chemical titration, the key mechanism is a reaction between aldehyde groups and hydroxylamine hydrochloride, which produces oxime compounds and hydrogen chloride [34,42]. This hydrogen chloride was titrated by sodium hydroxide and the DS was calculated by Equation (2).

(2) $$\mathrm{DS}=\;\frac{\mathrm{mol}\;\mathrm{of}\;\left(-\mathrm{CHO}\right)}{\mathrm{mol}\;\mathrm{of}\;\mathrm{AGU}}=\frac{{\mathrm{mol}\;\mathrm{of}\;\mathrm{Na}}^{+}}{\mathrm{mol}\;\mathrm{of}\;\mathrm{AGU}}=\frac{M_{{\mathrm{Na}}^{+}}\left(\mathrm{mol}/L\right)\;\times {\;V}_{{\mathrm{Na}}^{+}}\left(\mathrm{mL}\right)\;\times \;\frac{1\;L}{1000\;\mathrm{mL}}}{W_{\mathrm{sample}}\left(g\right)\;\times \;\frac{1\;\mathrm{AGU}}{162\;g/\mathrm{mol}}}$$

Table 3 showed the DS of starch aldehydes where DS was increased with increasing content of sodium periodate. Qualitative and quantitative analysis commonly showed that oxidant content has a close relationship to the aldehyde substitution of starch hydroxyl groups. However, when the DS difference according to starch source was analyzed, starch aldehydes based on corn starch showed higher DS compared to those based on potato starch. The main reason for the DS difference is that the amylopectin content of corn starch is higher than that of potato starch [43,44].

In previous studies, DS also increased as the amylopectin content increased, because amylopectin is more prone to oxidation than amylose [38,45]. In the case of hypochlorite oxidation, the carboxyl formation is likely to occur close to the branching points of short amylopectin chains where the degrees of polymerization are 6–12 and 13–24, respectively. As the amylopectin content increases, more short chains are present and lead to increased branching points [46]. In addition, these branching points lead to increased distance between the amylopectin and amylopectin/amylose chains, which makes it easier for oxidant to enter the starch hydroxyl groups [38].

### 3.3. Surface Morphology of Starch Aldehydes

**Figure 2:**
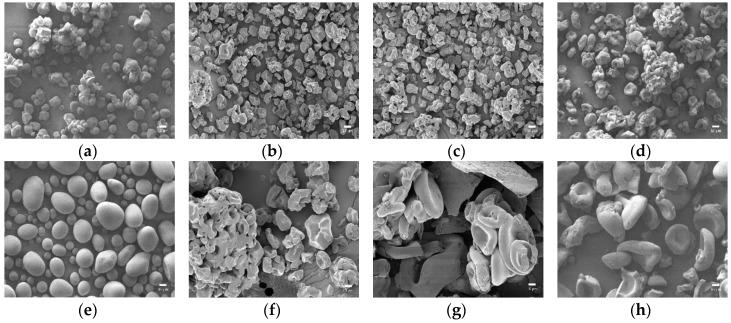
The FE-SEM images of native starch and starch aldehydes (×1000, scale bar: 10 μm): (**a**) CS; (**b**) CS10; (**c**) CS20; (**d**) CS30; (**e**) PS; (**f**) PS10; (**g**) PS20; (**h**) PS30.

In Figure 2, the particles of native starch presented spherical or elliptical shapes with smooth surfaces in sizes ranging from 10 to 20 μm for corn starch and 10 to 40 μm for potato starch. The particle sizes varied according to starch source, but the particle shape changed consistently after oxidation in both corn and potato starch. As the content of oxidant increased, the particles had a distortion with significant wrinkles since the cleavage of glucoside rings by oxidation led to an uneven surface, creating pits on the particles [39,47].

The particles of starch aldehydes obviously showed lima bean or hemoglobin-like shapes and showed that the interior of starch granules was easily corroded [40]. According to a previous study, the amorphous regions of starch are mainly located inside starch granules while the crystalline areas mainly exist on the outside [48]. Therefore, the lima bean or hemoglobin-like shapes of starch aldehyde particles indicated that the oxidation by periodate is more prone in the amorphous regions than in the crystalline areas. In addition, particles of starch aldehydes were conglomerated closely and the particles became bigger in contrast to the native starch. The agglomeration could be due to the strong depolymerization and the oxidation of surface, which lead to higher interactions between the granules [39,47].

### 3.4. Chemical Crosslinking of Starch Aldehydes-CMC SAPs

**Figure 3:**
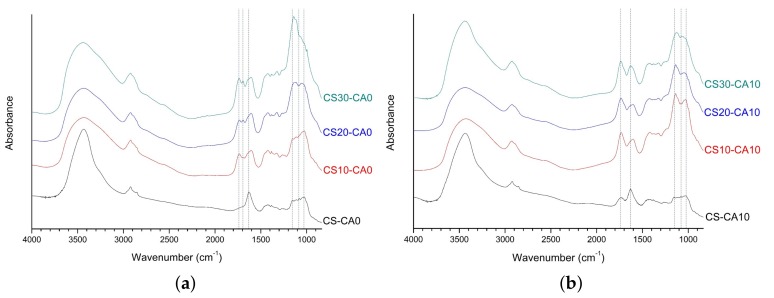
The FT-IR spectra of polysaccharide-based SAPs: (**a**) CS-CA0, CS10-CA0, CS20-CA0, CS30-CA0; (**b**) CS-CA10, CS10-CA10, CS20-CA10, CS30-CA10.

The FT-IR analysis was conducted to investigate the chemical crosslinking of prepared SAPs. First, as shown in Figure 3a, the spectra of the SAPs without citric acid were analyzed. The spectrum of CS-CA0 showed that native starch and CMC were physically mixed without chemical bonding as the major peaks of CMC and native starch were all found in CS-CA0. The bands at 1630 and 1421 cm^−1^ were derived from CMC, which were attributed to COO- stretching and CH_2_ absorption, respectively [23,24]. Additionally, the peaks at 1153, 1080, and 1025 cm^−1^ were derived from native starch, which were the characteristics of C–O stretching in anhydroglucose ring.

The FT-IR spectra of CS10-CA0, CS20-CA0, and CS30-CA0, which were prepared by the chemical crosslinking of starch aldehydes and CMC, showed the following peak characteristics. As expected, the peaks of unreacted starch aldehydes at 1735 cm^−1^ and the carboxyl group peaks of CMC at 1605 cm^−1^ were detected, respectively. A shift to the peak of 1605 cm^−1^ compared to the peak of 1630 cm^−1^ indicated that starch aldehydes and CMC may be involved in the crosslinking to form an acetal bridge. Generally, aldehyde is reacted with the hydroxyl group to form an acetal bridge at acidic pH [49], but it confirmed that a C=C linkage was formed due to a side reaction. Similar to the results of previous studies, the new peak at 1695 cm^−1^ was attributed to be the C=C double bond of aldol condensation products between starch aldehydes [50].

In the FT-IR analysis, the position of the acetal C-O-C band is similar to that of the fingerprint region for polysaccharides. Thus, it was necessary to analyze the spectra at 1200–1000 cm^−1^ for CS-CA0, CS10-CA0, CS20-CA0, and CS30-CA0 in order to confirm the formation of the acetal bridge. The peaks at 1153, 1080, and 1025 cm^−1^ for CS-CA0 and CS10-CA0 were no longer present in CS20-CA0 and CS30-CA0. Instead, the new peak appeared at 1140 cm^−1^. In other words, the C-O stretching at 1153 and 1080 cm^−1^ were replaced by the new absorption band, which could be attributed to C-O ether and C-O-C acetal bridge formed by CMC crosslinking with starch aldehydes. It was similar to previous studies where polyvinyl alcohol was crosslinked with glutaraldehyde [51,52]. Therefore, it was confirmed that starch aldehydes acted as crosslinking agents among polysaccharide chains.

Next, the spectra of the SAPs with citric acid were analyzed. As shown in Figure 3b, the new peak for CS-CA10 was observed at 1735 cm^−1^, which could be attributed to the characteristic stretching band of the carbonyl group related to anhydride formation [30,53]. It demonstrated that the crosslinking between the hydroxyl groups of the polysaccharides and citric acid was well formed. The peaks of CMC carboxyl groups at 1630 cm^−1^ and the peaks of starch anhydroglucose ring at 1153, 1080, and 1025 cm^−1^ still remained.

For CS10-CA10, CS20-CA10, and CS30-CA10 using starch aldehydes, the spectra showed a dramatic change. The spectrum of CS10-CA10 showed the ester linkage peak by citric acid at 1732 cm^−1^ and the peak shift of CMC carboxyl groups at 1604 cm^−1^. Looking at the peaks associated with the anhydroglucose ring, the peaks at 1153 and 1025 cm^−1^ shifted to 1137 and 1029 cm^−1^, respectively, while the peak at 1080 cm^−1^ weakened. For CS20-CA10 and CS30-CA10, the tendencies were same and these SAPs showed the two bands at 1137–1129 and 1063–1053 cm^−1^, respectively. Moreover, the C=O peak related to the ester bridging of polysaccharides and citric acid was more strongly expressed than the starch aldehydes-related peaks at 1735–1695 cm^−1^. The C-O peaks related to the acetal bridge by starch aldehydes and the ester bridge by citric acid were mixed at 1200–1000 cm^−1^.

**Figure 4:**
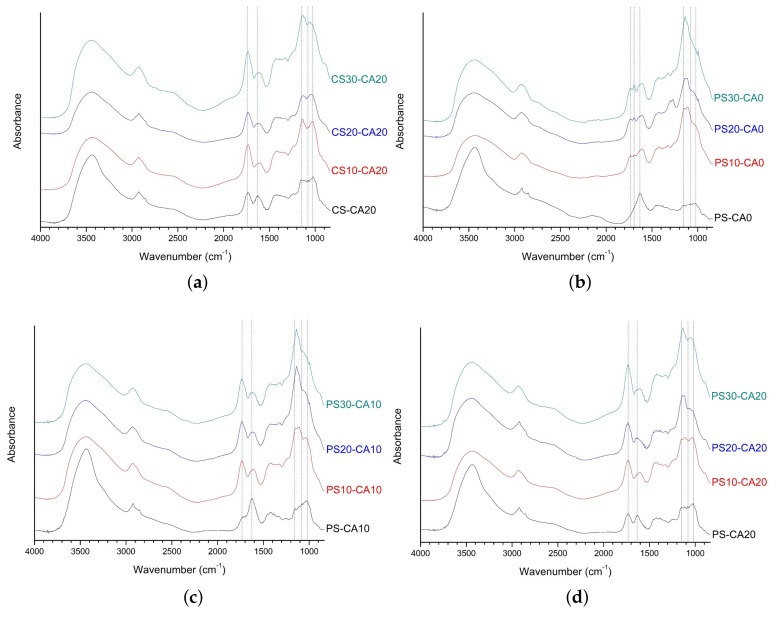
The FT-IR spectra of polysaccharide-based SAPs: (**a**) CS-CA20, CS10-CA20, CS20-CA20, CS30-CA20; (**b**) PS-CA0, PS10-CA0, PS20-CA0, PS30-CA0; (**c**) PS-CA10, PS10-CA10, PS20-CA10, PS30-CA10; (**d**) PS-CA20, PS10-CA20, PS20-CA20, PS30-CA20.

As shown in Figure 4a, the peak intensities of citric acid-related ester linkage for CS-CA20, CS10-CA20, CS20-CA20, and CS30-CA20 were stronger at 1733 cm^−1^. The positions of other main bands were the same as the results in the above-mentioned analysis. In the FT-IR analysis of all SAPs using potato starch aldehydes (Figure 4b–d), the tendencies of the spectra were similar to those of SAPs using corn starch aldehydes. It was slightly different that the 1140 cm^−1^ peak intensity associated with the acetal bridge was stronger compared to the results of corn starch aldehydes.

### 3.5. Swelling Property of Starch Aldehydes-CMC SAPs

The swelling behavior of prepared SAPs was analyzed by several parameters. First, the effect of starch aldehydes and citric acid on the equilibrium swelling ratio was confirmed. The equilibrium swelling ratio was calculated by measuring the swelling ratio after immersing the SAPs in water for 24 h. In exceptional cases, the CS-CA0 (maximum 21.0 g/g) and the PS-CA0 (maximum 58.4 g/g) with only physical interactions showed the highest swelling ratios after 8 h of swelling. It was followed by the gel degradation and the decrease of the swelling ratio.

**Figure 5:**
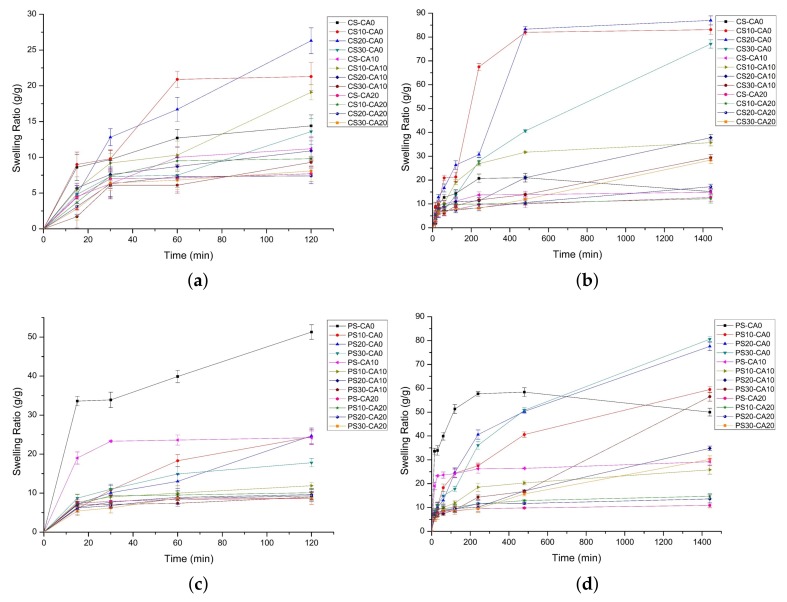
The swelling ratios of polysaccharide-based SAPs using corn starch series (**a**,**b**) and potato starch series (**c**,**d**): the initial swelling in 2 h (**a**,**c**) and the whole swelling in 24 h (**b**,**d**) (mean ± SD, *n* = 5).

In Figure 5a,b, the equilibrium swelling ratios of the SAPs using native corn starch were from 12.7 (CS-CA20) to 15.2 g/g (CS-CA0) while using corn starch aldehydes were from 12.2 (CS10-CA20) to 87.0 (CS20-CA0) g/g. It is due to the loose network structure between starch aldehydes and CMC by the acetal bridge rather than the dense structure between native starch and CMC. A large amount of water enters the network structure of the SAPs and the swelling ratio increases as water is caught by the interaction with the CMC carboxyl groups. The CS30-CA0 (77.1 g/g) and the CS30-CA10 (29.4 g/g) had lower equilibrium swelling ratios than the CS20-CA0 (87.0 g/g) and the CS20-CA10 (37.8 g/g) since starch aldehydes with very high DS produce more crosslinking and interfere with water penetration. The effect of citric acid on the equilibrium swelling ratio was also confirmed. Regardless of the type of starch or starch aldehydes used in SAP preparation, the equilibrium swelling ratio decreased when increasing citric acid content, because the ester bridge combined by citric acid with polysaccharides forms strong crosslinking and interferes with water penetration.

In Figure 5c,d, the tendencies of the equilibrium swelling ratios for SAPs using native potato starch or starch aldehydes were similar to using native corn starch or starch aldehydes, but there were some differences. The equilibrium swelling ratios of PS-CA0 (50.0 g/g) and PS-CA10 (29.2 g/g) were higher than those of CS-CA0 (15.2 g/g) and CS-CA10 (14.9 g/g). As starch undergoes gelatinization at 100 °C and retrodegradation at 55 °C in SAP preparation, the disaggregated starch chains retrograde gradually into partially ordered structures and form a 3-dimensional network. In particular, the amylose chains form hydrogen bonds [54]. Therefore, according to the calculation of starch aldehyde DS described above, the amylose content of potato starch is considered to be high, so that the equilibrium swelling ratios of PS-CA0 and PS-CA10 are considered to be high.

The SAPs using PS10 (maximum 59.5 g/g) and PS20 (maximum 77.6 g/g) starch aldehydes had lower equilibrium swelling ratios than the SAPs using CS10 (maximum 83.1 g/g) and CS20 (maximum 87.0 g/g) starch aldehydes. Because of the relatively low DS, the acetal bridges were less formed. Interestingly, the CS20-CA0 (87.0 g/g) showed the highest equilibrium swelling ratio in corn starch series while the PS30-CA0 (80.6 g/g) showed the highest equilibrium swelling ratio in potato starch series. It means that there is a slight difference in reactivity and crosslinking density depending on the starch source. In addition, the SAPs using PS30 starch aldehyde showed the significant equilibrium swelling ratios (from 30.2 to 80.6 g/g) even when citric acid was added. It is considered that the equilibrium swelling ratio is increased when the acetal bridges due to starch aldehydes are higher than the ester bridges due to citric acid.

Next, to analyze the swelling behavior in more detail, two swelling kinetic models were applied. The initial diffusion of water molecules into the voids of SAPs could be analyzed by the Fickian diffusion model (Equation (3)). The entire swelling process by chain relaxation was evaluated by the Schott’s pseudo second order kinetics model (Equation (4)) [19,55].

(3) $$F=\frac{W_{t}}{W_{eq}}=Kt^{n},\ln\left(F\right)=\ln(\frac{W_{t}}{W_{eq}})=\ln K+n\ln t$$

(4) $$\frac{t}{Q_{t}}=\frac{1}{k_{is}}+\frac{t}{Q_{\infty }}=\frac{1}{k_{s}Q_{\infty }^{2}}+\frac{t}{Q_{\infty }}$$

In the Fickian diffusion model, *F* is the fractional uptake at the given time *t* (min) while *W_t_* and *W_eq_* are the weight (g) of absorbed water at time *t* and equilibrium, respectively. *K* is the proportionality constant of SAPs and *n* describes the type of diffusion mechanism. In the Schott’s pseudo second order kinetics model, *Q_t_* (g/g) is the swelling ratio of SAPs at time *t*, $Q_{\infty }$ (g/g) is the theoretical equilibrium swelling ratio, *k_is_* ((g/g)/min) is the initial swelling rate constant, and *k_s_* ((g/g)/min) is the swelling rate constant.

Each SAP was slightly different in the detailed swelling behavior, but overall swelling behavior involved the following steps. As shown in Table 4, the Fickian diffusion model confirmed the initial swelling behavior and had two characteristics. The first feature was that the SAPs used only starch aldehydes without citric acid showed the non-Fickian diffusion (*n* is between 0.5 and 1.0) while other SAPs followed the Fickian diffusion (*n* is less than 0.5) [19]. The non-Fickian diffusion SAPs showed that the initial water absorption was controlled collaboratively by water diffusion and relaxation of polymer chains [55]. It was followed by the high equilibrium swelling ratios (*Q_eq_*, from 77.1 to 87.0 g/g). However, the Fickian diffusion SAPs showed the relatively low equilibrium swelling ratios (from 10.9 to 56.5 g/g).

The second feature was that the Fickian diffusion model with good correlation coefficient (*R*^2^ > 0.9) could be applied to the SAPs using starch aldehydes with low DS. It was because the SAPs using starch aldehydes with low DS had the initial swelling in 15 to 30 min, and after that, it maintained a constant level of swelling less than 2 h. However, the SAPs using starch aldehydes with high DS were swollen additionally from 30 min to 120 min after the initial swelling. As a result, an accurate plot of ln(*W_t_*/*W_eq_*) as function of ln(*t*) could not be obtained.

In the Schott’s pseudo second order kinetics model, the theoretical equilibrium swelling ratio, the initial swelling rate constant, and the swelling rate constant obtained from the plots of *t*/*Q_t_* versus *t* are given in Table 5. Similar to the plotting results of the Fickian diffusion model, the SAPs using starch aldehydes with low DS showed straight lines with high correlation coefficients (*R*^2^ > 0.9). The SAPs plotted with low correlation coefficients were characterized by increased swelling between 8 and 24 h after the start of swelling, so it was difficult to calculate the exact swelling parameters. In spite of this mismatch, the theoretical equilibrium swelling ratios matched the experimental data, which indicated that the swelling process followed the Schott’s pseudo second order kinetics model. In addition, the relationship between the initial swelling rate constant and the equilibrium swelling ratio was analyzed. Although the tendencies of all SAPs were not perfect, the initial swelling rate constant decreased as the DS of starch aldehydes or the content of citric acid increased.

Interestingly, the reduced initial swelling rate constant by adding citric acid resulted in decreased equilibrium swelling ratio. However, the decreased initial swelling rate constant using starch aldehydes increased the equilibrium swelling ratio. The latter case was prominent when measuring the swelling ratio of CS10-CA0, CS20-CA0, CS30-CA0, PS10-CA0, PS20-CA0, and PS30-CA0 SAPs. This phenomenon can be related to the aforementioned Fickian diffusion model. These SAPs are relatively active in water diffusion and polymer chain relaxation through the non-Fickian diffusion behavior. In other words, when a certain period of time has elapsed since the water diffused, it reaches a high equilibrium swelling ratio accompanied by rapid swelling around the acetal bridge. On the other hand, the SAPs added citric acid had low equilibrium swelling ratios, because the crosslinking density was dense and the polysaccharide chains could not freely expand.

### 3.6. Surface Morphology of Starch Aldehydes-CMC SAP

**Figure 6:**
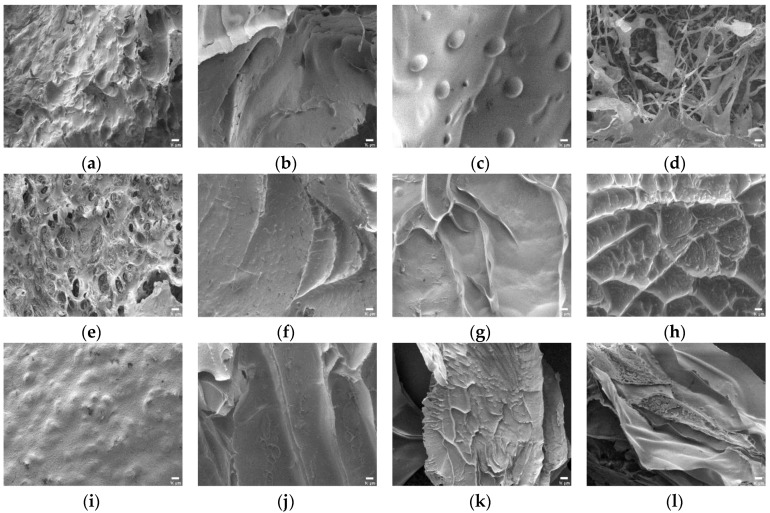
The FE-SEM images of polysaccharide-based SAPs (×1000, scale bar: 10 μm): (**a**) CS-CA0; (**b**) CS10-CA0; (**c**) CS20-CA0; (**d**) CS30-CA0; (**e**) CS-CA10; (**f**) CS10-CA10; (**g**) CS20-CA10; (**h**) CS30-CA10; (**i**) CS-CA20; (**j**) CS10-CA20; (**k**) CS20-CA20; (**l**) CS30-CA20.

Prior to obtaining the FE-SEM images, the swollen SAPs lyophilized to eliminate moisture. The surface morphology of the SAPs without citric acid were analyzed first. In Figure 6a, the image of CS-CA0 showed granular aggregates with a convex surface. Since it was not a dense network shape, these structures were considered disadvantageous in retaining a large amount of water. However, in Figure 6b–d, the images of CS10-CA0, CS20-CA0, and CS30-CA0 using starch aldehydes showed that coarse and broad network structures were present instead of spherical aggregation. In addition, it was confirmed that the pore sizes of these SAPs were different. The CS10-CA0 had small pores, but the CS20-CA0 had remarkably circular pores. In particular, the CS30-CA0 showed a porous morphology, which was confirmed to be a favorable material for absorbing water [16,21].

**Figure 7:**
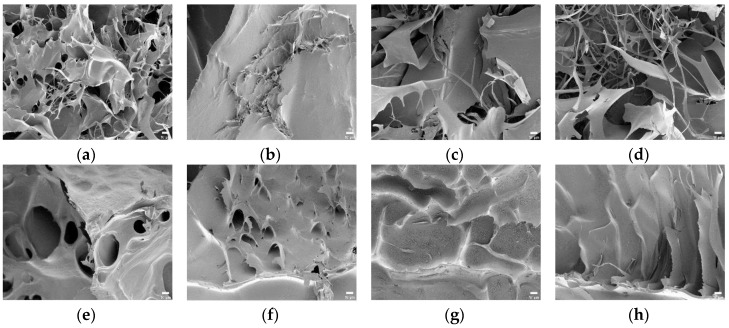

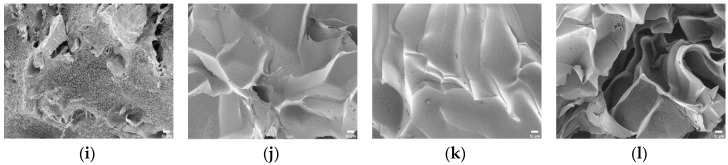
The FE-SEM images of polysaccharide-based SAPs (×1000, scale bar: 10 μm): (**a**) PS-CA0; (**b**) PS10-CA0; (**c**) PS20-CA0; (**d**) PS30-CA0; (**e**) PS-CA10; (**f**) PS10-CA10; (**g**) PS20-CA10; (**h**) PS30-CA10; (**i**) PS-CA20; (**j**) PS10-CA20; (**k**) PS20-CA20; (**l**) PS30-CA20.

In Figure 7a–d, the surface morphology of the SAPs with potato starch aldehydes was different from that of the SAPs using corn starch aldehydes and all samples showed coarse and broad network structures. The PS-CA0 had many pores and absorbed a lot of water in contrast to the CS-CA0. The PS10-CA0 showed small pore sizes while porous morphology was showed in the PS20-CA0 and the PS30-CA0. When the results were summarized, the SAPs without citric acid showed a network structure in all samples except the CS-CA0. It also coincided with the aforementioned high equilibrium swelling ratio results.

Furthermore, the changes in SAP morphology by adding citric acid were analyzed. In general, the images of SAPs showed various types of particles with irregular distribution. In particular, the addition of starch aldehydes had a significant effect on the surface morphology of the SAPs. In Figure 6e, the CS-CA10 showed similar surface morphology to the CS-CA0 where the roughness became stronger. On the other hand, in Figure 6f–h, the CS10-CA10, the CS20-CA10, and the CS30-CA10 containing starch aldehydes had wrinkles on the surface. As the DS of starch aldehydes increased, the wrinkles also increased. These wrinkles lead to a higher specific surface area for the SAPs and may be regions where water can interact with the hydrophilic groups of SAPs, which facilitate the permeation of water into the polymeric network [19]. However, when compared to the SAPs without citric acid, it showed a semi-porous structure with relatively small pores. It was a decisive factor in preventing large amounts of water absorption. Moreover, in Figure 7e–h, the obtained SAP images from the potato starch series were similar to the aforementioned descriptions.

When more citric acid is added into the SAPs, inevitably more crosslinking is formed. Therefore, it was necessary to analyze whether this phenomenon affected the surface morphology. As shown in Figure 6i–l and Figure 7i–l, no significant difference was observed in comparison to the SAPs with low citric acid content. However, looking at the surface curvature and void in detail, the surface was slightly smoother and the pore size was reduced.

## 4. Conclusions

CMC was used as a main material for the production of polysaccharide-based SAPs. Starch aldehydes and citric acid were used as crosslinking agents to make the polymer network structure, which is characteristic of the SAPs. Starch aldehydes were prepared by oxidizing corn and potato starch with sodium periodate. The FT-IR analysis of starch aldehydes revealed the appearance of characteristic aldehyde peak at 1732 cm^−1^ and the disruption of anhydroglucose ring. The hydroxyl groups of C-2 and C-3 of the anhydroglucose ring were successfully substituted with the aldehyde groups and the DS changed according to the amount of sodium periodate and the source of starch. The DS of corn starch aldehydes was higher than that of potato starch aldehydes and the average maximum DS was about 2.48. The FE-SEM image confirmed that the morphology of starch aldehydes was changed from the elliptical native starch shape to the hemoglobin-like shape.

The crosslinking reaction between starch aldehydes and CMC formed the network structure of polysaccharide-based SAPs. When citric acid was introduced, an ester linkage was additionally formed in polysaccharide-based SAPs. Through FT-IR analysis and swelling ratio measurement of the SAPs, the acetal bridges by starch aldehydes increased water uptake, but the ester bridges by citric acid had the opposite effect. Among the various SAPs, the highest equilibrium swelling ratio was found in CS20-CA0 and PS30-CA0 depending on the starch source where the values were 87.0 g/g and 80.6 g/g, respectively. In addition, the initial swelling behavior and the entire swelling process for all SAPs was analyzed by the Fickian diffusion model and the Schott’s pseudo second order kinetics model, respectively. Finally, the difference in equilibrium swelling ratio was determined by presence and distribution of the porous structures in FE-SEM images.

In summary, the bio-based components were introduced to prepare the SAPs with excellent crosslinking and high water absorption. The materials developed in this experiment may be good candidates for environment-friendly SAP products in the future.

## Figures and Tables

**Table 1:** The ingredient compositions that were used to prepare starch aldehydes.

Sample Code	Composition	Sample Code	Composition
CS	Corn starch 24.3 g	PS	Potato starch 24.3 g
CS10	CS + NaIO_4_ 10.69 g	PS10	PS + NaIO_4_ 10.69 g
CS20	CS + NaIO_4_ 21.38 g	PS20	PS + NaIO_4_ 21.38 g
CS30	CS + NaIO_4_ 32.07 g	PS30	PS + NaIO_4_ 32.07 g

**Table 2:** The ingredient compositions that were used to prepare polysaccharide-based superabsorbent polymers (SAPs).

Sample Code	Composition	Sample Code	Composition
CS-CA0	CS + CMC	PS-CA0	PS + CMC
CS10-CA0	CS10 + CMC	PS10-CA0	PS10 + CMC
CS20-CA0	CS20 + CMC	PS20-CA0	PS20 + CMC
CS30-CA0	CS30 + CMC	PS30-CA0	PS30 + CMC
CS-CA10	CS + CMC + Citric acid 10%	PS-CA10	PS + CMC + Citric acid 10%
CS10-CA10	CS10 + CMC + Citric acid 10%	PS10-CA10	PS10 + CMC + Citric acid 10%
CS20-CA10	CS20 + CMC + Citric acid 10%	PS20-CA10	PS20 + CMC + Citric acid 10%
CS30-CA10	CS30 + CMC + Citric acid 10%	PS30-CA10	PS30 + CMC + Citric acid 10%
CS-CA20	CS + CMC + Citric acid 20%	PS-CA20	PS + CMC + Citric acid 20%
CS10-CA20	CS10 + CMC + Citric acid 20%	PS10-CA20	PS10 + CMC + Citric acid 20%
CS20-CA20	CS20 + CMC + Citric acid 20%	PS20-CA20	PS20 + CMC + Citric acid 20%
CS30-CA20	CS30 + CMC + Citric acid 20%	PS30-CA20	PS30 + CMC + Citric acid 20%

**Table 3:** The DS of starch aldehydes (mean ± SD, *n* = 5).

Sample Code	CS10	CS20	CS30	PS10	PS20	PS30
**DS**	1.01 ± 0.03	1.84 ± 0.16	2.47 ± 0.30	0.76 ± 0.17	1.59 ± 0.06	2.48 ± 0.56

**Table 4:** The swelling parameters of polysaccharide-based SAPs in the Fickian diffusion model.

Sample Code	*n*	*k*	*R*^2^	Sample Code	*n*	*k*	*R*^2^
CS-CA0	0.258	0.185	0.951	PS-CA0	0.219	0.270	0.939
CS10-CA0	0.701	0.011	0.922	PS10-CA0	0.552	0.026	0.912
CS20-CA0	0.643	0.010	0.894	PS20-CA0	0.653	0.012	0.957
CS30-CA0	0.624	0.007	0.888	PS30-CA0	0.492	0.022	0.884
CS-CA10	0.408	0.107	0.945	PS-CA10	0.027	0.771	0.940
CS10-CA10	0.523	0.039	0.934	PS10-CA10	0.287	0.127	0.902
CS20-CA10	0.284	0.067	0.971	PS20-CA10	0.135	0.127	0.910
CS30-CA10	0.344	0.054	0.832	PS30-CA10	0.255	0.042	0.739
CS-CA20	0.128	0.389	0.986	PS-CA20	0.078	0.565	0.928
CS10-CA20	0.128	0.409	0.943	PS10-CA20	0.070	0.474	0.847
CS20-CA20	0.234	0.140	0.915	PS20-CA20	0.210	0.269	0.946
CS30-CA20	0.181	0.106	0.858	PS30-CA20	0.272	0.082	0.821

**Table 5:** The swelling parameters of polysaccharide-based SAPs in the Schott’s pseudo second order kinetics model.

Sample Code	*Q_eq_* (Mean ± SD, *n* = 5)	*Q_∞_*	*k_is_*	*k_s_*	*R*^2^
CS-CA0	15.2 ± 1.16	22.9	0.541	$1.03\times 10^{-3}$	0.989
CS10-CA0	83.1 ± 1.95	98.2	0.446	$4.62\times 10^{-5}$	0.940
CS20-CA0	87.0 ± 1.87	108.5	0.321	$2.73\times 10^{-5}$	0.911
CS30-CA0	77.1 ± 1.76	105.8	0.172	$1.54\times 10^{-5}$	0.843
CS-CA10	14.9 ± 1.56	15.3	0.407	$1.74\times 10^{-3}$	0.999
CS10-CA10	35.7 ± 1.38	38.6	0.338	$2.27\times 10^{-4}$	0.997
CS20-CA10	37.8 ± 1.38	43.0	0.137	$7.40\times 10^{-5}$	0.828
CS30-CA10	29.4 ± 1.34	33.7	0.100	$8.86\times 10^{-5}$	0.866
CS-CA20	12.7 ± 1.55	13.0	0.203	$1.20\times 10^{-3}$	0.992
CS10-CA20	12.2 ± 1.83	12.4	0.311	$2.03\times 10^{-3}$	0.996
CS20-CA20	17.2 ± 1.09	18.1	0.117	$3.55\times 10^{-4}$	0.944
CS30-CA20	28.4 ± 1.54	31.7	0.089	$8.84\times 10^{-5}$	0.729
PS-CA0	50.0 ± 1.64	61.3	2.815	$7.50\times 10^{-4}$	0.998
PS10-CA0	59.5 ± 1.24	66.2	0.315	$7.20\times 10^{-5}$	0.973
PS20-CA0	77.6 ± 1.78	91.4	0.312	$3.74\times 10^{-5}$	0.975
PS30-CA0	80.6 ± 1.01	96.4	0.286	$3.07\times 10^{-5}$	0.929
PS-CA10	29.2 ± 1.42	29.4	1.377	$1.59\times 10^{-3}$	0.999
PS10-CA10	25.8 ± 1.82	27.1	0.270	$3.66\times 10^{-4}$	0.990
PS20-CA10	34.8 ± 0.97	38.8	0.122	$8.07\times 10^{-5}$	0.776
PS30-CA10	56.5 ± 1.88	67.8	0.110	$2.39\times 10^{-5}$	0.450
PS-CA20	10.9 ± 1.04	11.0	0.426	$3.52\times 10^{-3}$	0.998
PS10-CA20	14.7 ± 1.07	15.0	0.344	$1.53\times 10^{-3}$	0.997
PS20-CA20	13.6 ± 1.70	13.9	0.313	$1.63\times 10^{-3}$	0.997
PS30-CA20	30.2 ± 1.45	33.5	0.115	$1.02\times 10^{-4}$	0.824
